# Mycotoxins are a component of *Fusarium graminearum* stress-response system

**DOI:** 10.3389/fmicb.2015.01234

**Published:** 2015-11-04

**Authors:** Nadia Ponts

**Affiliations:** UR1264 - MycSA, Institut National de la Recherche Agronomique, Centre de Bordeaux-AquitaineVillenave d'Ornon, France

**Keywords:** mycotoxins, adaptation, stress-response system, DON, *Fusarium graminearum*

## Secondary metabolites in the context of phytopathogenic fungal life

*Fusarium graminearum* is a phytopathogenic Ascomycota that can cause Fusarium head blight in wheat and other cereals worldwide, leading to important yield losses as well as reduced grain quality. The analysis of the *F. graminearum* genome sequence revealed the presence of 20 non-ribosomal peptide synthases, 15 polyketide synthases, and 17 terpenoid synthases, all potentially involved in the production of a panel of secondary metabolites, including yet unknown ones, such as the mycotoxins deoxynivalenol (DON) and other type B trichothecenes (Cuomo et al., 2007). The toxicity of DON to humans and animals upon ingestion has been extensively illustrated (see Bonnet et al., 2012; Awad et al., 2013; Pinton and Oswald, 2014 for reviews), and the presence of this mycotoxin in cereal-derived food and feeds represents a serious threat for public health (reviewed in Marin et al., 2013; Sirot et al., 2013; Wu et al., 2014).

Secondary metabolites are a structurally diverse family of compounds that are often qualified as unessential for short-term development but important for long-term survival (see Roze et al., 2011a for a review). Secondary metabolism is tightly linked to primary metabolism, starting with the fact that primary metabolites “feed” secondary metabolite biosynthetic pathways (reviewed in Audenaert et al., 2014; Sheridan et al., 2015). For example, the biosynthesis of type B trichothecenes by *F. graminearum* derives from the isoprenoid pathway, an essential metabolic pathway involved in various cellular processes. Remarkably, the genes involved in the biosynthesis of isoprenoids are positively regulated by the transcription factor Tri6, which also regulates the genes involved in the trichothecenes biosynthetic pathway (Seong et al., 2009). Moreover, Menke et al. (2013) proposed that type B trichothecenes are produced in specific cellular vesicles, so-called “*toxisomes*,” and found pieces of evidence that the HMG-CoA reductase of the isoprenoid pathway also localizes to toxisomes when the production of toxins is induced (Menke et al., 2013). Whether these toxisomes are neo-formed for toxin biosynthesis or derive from vesicles hosting elements of the primary metabolism remains to be investigated. Nonetheless, the production of mycotoxins by *F. graminearum* could be the result of a tight coordination with primary metabolism.

Fungi are known to produce diverse families of secondary metabolites, biological functions of which are not yet fully understood. In the context of host-pathogen interactions, DON was shown to be a virulence factor for *F. graminearum* infecting wheat by promoting the spreading of the pathogen (Jansen et al., 2005). During infection and colonization, *F. graminearum* is particularly exposed to plant metabolites that can be constitutive components of the host or molecules produced as a consequence of the presence of the pathogen. Some of these metabolites can trigger fungal stress-response pathways. There are accumulated evidences showing that the production of fungal secondary metabolites, DON in particular, could be an element of the general stress response in *F. graminearum*.

## Secondary metabolites are parts of stress response pathways

Stresses caused by variations of the environment can be biotic (the surrounding microbiome) or abiotic (*e.g*., heat, pH, light, *etc*.), and both types can lead to either adaptation and survival, or cell death. Here, cell death does not necessary mean a failure to implement an effective stress response but can be the result of the response pathway itself when a destructive response is implemented. The effects of various stresses that phytopathogenic fungi are likely to encounter in the context of host invasion have been the subjects of numerous works. In particular, oxidative stress, as the result of the early defensive “oxidative burst” triggered in the host plant upon infection, has been intensively examined.

### Oxidative stress

Oxidative stress has been extensively studied regarding its interaction with secondary metabolism in fungi. Secondary metabolites can counteract or, conversely, enhance the deleterious effects of oxidative stress, and fungi may use reactive oxygen species (ROS) as signals that initiate/modulate biosynthesis (reviewed in Hong et al., 2013a; Montibus et al., 2015; Sheridan et al., 2015). Response to oxidative stress and fungal secondary metabolism are indeed intertwined. Many of the regulators involved in such response described to date belong to the basic leucine zipper (bZIP) family of transcription factors as illustrated below.

In *Aspergillus nidulans*, the Yap-like bZIP factor *NapA*, was shown to be involved in tolerance to oxidative stress (Asano et al., 2007). Yin et al. (2013) showed that treatment with the pro-oxidant tert-butyl hydroperoxide (tBOOH) is associated with increased accumulation of the mycotoxin sterigmatocystin, an effect counteracted by over-expressing *NapA* (Yin et al., 2013). A similar trend was observed in the ochratoxin producer *Aspergillus ochraceus* and the *NapA* orthologue *AoYap1* (Reverberi et al., 2012). Not only is ochratoxin production no longer stimulated by tBOOH in a mutant disrupted for *AoYap1* but toxin accumulation is also enhanced in untreated conditions (Reverberi et al., 2012).

On the mechanistic side, mobility shift assays provided experimental evidence that antioxidant and aflatoxin biosynthetic genes in *Aspergillus parasiticus* have binding sites for the same bZIP transcription factor *AtfB* that is activated upon oxidative stress *via* MAPK signaling (Hong et al., 2013a,b). In addition, chromatin immunoprecipitation assays showed that the binding of AtfB on the promoters of aflatoxin genes occurs only when *A. parasiticus* is grown in toxin-inducing medium (Roze et al., 2011b), which supports the concept that toxin biosynthesis and oxidative stress are tightly linked. Considering the interconnectivity between oxidative stress response and mycotoxin production illustrated above, mycotoxin production as a way to cope with endogenous oxidative stress has been previously proposed in *Aspergillus* species (see Reverberi et al., 2010 for a review). In the gray mold fungus *Botrytis cinerea*, the *BcAtf1* factor was also shown to positively regulate the production of secondary metabolites (Temme et al., 2012).

In *F. graminearum*, previous results indicate that oxidative stress with H_2_O_2_ could be a pre-requisite for the biosynthesis of the mycotoxin DON, which may suggest that DON production and endogenous oxidative stress could be connected (Ponts et al., 2006, 2007; Montibus et al., 2013). In this case, stress response and the regulation of DON synthesis are mediated by the transcription factor *FgAP1* that activates the transcription of antioxidant enzymes (Montibus et al., 2013). Possible sources of endogenous oxidative stress in fungi include NAPDH oxidases (Nox) that generate superoxide anions. However, gene deletion experiments showed that the characterized NADPH oxidases NoxA and NoxB do not seem to play a role in the production of trichothecenes (Wang et al., 2014). Other sources of ROS such as mitochondrial respiration or monoamine oxidase could be involved. Similarly, the regulator of transcription FgSKN7 seem to be involved in both oxidative and cell wall stress response, as well as DON biosynthesis (Jiang et al., 2015). All together, it seems likely that the production of DON and its acetylated derivatives is part of an adaptive response to oxidative stress (reviewed in Audenaert et al., 2014; Montibus et al., 2015). In this context, the ROS produced by the host plant as an early defense mechanism in response to infection are used by *F. graminearum* to its own benefit. Recent data may bring clues as of the mechanism of activation of DON biosynthesis upon oxidative stress. A glycogen synthase kinase GSK3 was shown to be essential for both virulence and DON production *F. graminearum*, and to be up regulated upon oxidative stress by H_2_O_2_ (Qin et al., 2015). These considerations strongly suggest that mycotoxin biosynthesis and response to oxidative stress are intertwined.

### Other stresses

The pH of the environment was shown to be particularly critical for the initiation of trichothecene B biosynthesis in *F. graminearum*. An acidic pH is a prerequisite for DON production (Merhej et al., 2010). The biosynthesis of type B trichothecenes has been shown to be negatively controlled by the transcription regulator *FgPac1*, homologous to the member of the pH regulator system *PacC* in *A. nidulans* (Merhej et al., 2011). It is noteworthy that oxidative stress and acidification have been described to occur together in various organisms including fungi (see examples in Jia et al., 2014; Pérez-Sampietro and Herrero, 2014). The relationship between these two stresses is however not clear.

Different response pathways can be triggered to counteract stresses that modify the organization and stability of the fungal cell wall. For example, the cell wall integrity pathway is responsive to changes in osmotic pressure and oxidative stress (see Hayes et al., 2014 for a review). Typically, the activation of the cell wall integrity pathway leads to the activation of cell wall biogenesis genes. In *F. graminearum*, the cell wall integrity pathway involves the MAP kinase FgMgv1 (Hou et al., 2002), itself activated by the MAP kinase FgMkk1 (Yun et al., 2014). When the pathway is altered by gene deletion, the production of DON is drastically reduced (Hou et al., 2002; Yun et al., 2014). Once more, the production of DON seems linked to a stress response pathway. It is noteworthy that the production of the secondary metabolite aurofusarin, a pigment, is also diminished in the absence of FgMgv1 (Hou et al., 2002). Such observation provides another example of the association between the activation of secondary metabolites pathways and stress response pathways. Remarkably, the MAP kinase FgOS-2 of the high osmolarity glycerol (HOG) response pathway is also activated by FgMkk1 (Yun et al., 2014), which illustrates the coupling between cell wall stress response and osmotic stress response. The HOG response pathway was initially described in the budding yeast as required for osmoadaptation (reviewed by Hohmann et al., 2007). In *F. graminearum*, FgHOG1 of the osmoregulation MAP kinase pathway, mediated by the FgOS-2 kinase, is involved in hyperosmotic as well as cell membrane stress responses. FgHOG1 also plays a role in ROS-mediated signaling. Its deletion causes a drastic reduction of DON accumulation, also caused by hyperosmotic conditions, as well as other developmental defects (Van Thuat et al., 2012; Zheng et al., 2012). Similarly, gene deletion experiments targeting FgOS-2 showed that DON production is strongly reduced *in planta* (Van Thuat et al., 2012).

## A secondary metabolism regulatory pathway part of environmental stress response

Regulators of fungal secondary metabolite biosynthesis that play a role in regulating other aspects of fungal life, including response to stress, have been described. The example of the *F. graminearum*, transcription factor Tri6 has been evoked above (Seong et al., 2009). In *Aspergilli*, LaeA has been described as a secondary metabolism-specific regulator involved in switching from inactive heterochromatin to the transcriptionally permissive euchromatic state. In *F. graminearum*, FgLae1 is a regulator of secondary metabolism that activates the production of DON (Kim et al., 2013). The deletion of *FgLae1* has pleiotropic effects in *F. graminearum*, also affecting sexual development for example (Kim et al., 2013). Typically LaeA is a member of the Velvet complex, involved in the response to light stress. In the absence of light, LaeA is available in the nucleus and chromatin at secondary metabolite gene clusters is active (and DON is produced for example). On the contrary, when light is applied, chromatin inactivates and secondary metabolite biosynthesis is shut down. Such a “secondary metabolism switch” could be conveniently associated with general stress response pathways to extend them to the production of secondary metabolites.

Previous observations made about *F. graminearum*'s response to oxidative stress, *i.e*., defensive plant-produced H_2_O_2_ may serve as a signal to produce DON (Ponts et al., 2006, 2007; Montibus et al., 2013), also fit with this theory. Other examples of such hijacking have been proposed, for example the use of the plant carbon and nitrogen metabolisms by *F. graminearum* for its own development and secondary metabolism (Audenaert et al., 2014). Polyamines such as agmatine or putrescine are, indeed, excellent activators of DON biosynthesis (Gardiner et al., 2010). From the evolutionary point of view, the general components of oxidative, osmotic, and cell wall stress pathways appear well conserved among fungi (Nikolaou et al., 2009). However, specific sensors and regulators involved in those pathways are diverse and seem to have fairly recently rapidly evolved to adapt to fungal specific life traits (Nikolaou et al., 2009). In a general manner, plant pathogens are more sensitive to oxidative stress than human pathogens for example (Nikolaou et al., 2009). Along the same line, stress response pathways in fungal plant pathogens, especially oxidative, osmotic and cell wall stress, are typically involved in tolerance to fungicides targeting the fungal cell wall (*e.g*., caspofungin, nikkomycin Z, tunicamycin, fluconazole; see Hayes et al., 2014 for a review). Such aspects must definitely be considered and addressed by strategies aiming at controlling mycotoxin occurrence in cereals.

The hypothesis of a coupling between stress response and mycotoxin production is reinforced by recent evidence that proteins involved in secondary metabolite pathways, including the aflatoxin one in *A. parasiticus*, co-localize with stress response proteins to the endosome/transport vesicles/vacuoles fraction of a fungal cell extract (Linz et al., 2012). In *F. graminearum*, previous work showed that the endoplasmic reticulum stress response and oxidative stress are tightly linked (Malhotra et al., 2008). Remarkably, recent work found that vesicular sequestration of enzymes of the DON-producing pathway co-localize with the endoplasmic reticulum (Menke et al., 2013; Boenisch et al., 2015). When all elements are taken into consideration, *F. graminearum* secondary metabolism and stress response pathways are indisputably very closely interconnected. Although further investigation is required, an attractive hypothesis is that secondary metabolism pathways could be part of the fungus' stress-response system. Under this scenario, more than a coupling of pathways, the production of DON and other secondary metabolites would be integral part of the fungus' arsenal to cope and adapt to its always-changing environment, including in the context of host-pathogen exchanges.

### Conflict of interest statement

The author declares that the research was conducted in the absence of any commercial or financial relationships that could be construed as a potential conflict of interest.
